# Retinal Nerve Fiber and Optic Disc Morphology in Patients with Human Immunodeficiency Virus Using the Heidelberg Retina Tomography 3

**DOI:** 10.1371/journal.pone.0133144

**Published:** 2015-08-10

**Authors:** Dirk-Uwe Bartsch, Igor Kozak, Igor Grant, Victoria L. Knudsen, Robert N. Weinreb, Byung Ro Lee, William R. Freeman

**Affiliations:** 1 Jacobs Retina Center at the Shiley Eye Institute, University of California San Diego, La Jolla, California, United States of America; 2 HIV Neurobehavioral Research Center (HNRC), San Diego, California, United States of America; 3 Retina Associates of Utah, Salt Lake City, Utah, United States of America; 4 Hamilton Glaucoma Center at the Shiley Eye Institute, University of California San Diego, La Jolla, California, United States of America; 5 Hanyang University, Seoul, South Korea; 6 King Khaled Eye Specialist Hospital, Riyadh, Saudi Arabia; Casey Eye Institute, UNITED STATES

## Abstract

**Purpose:**

To use novel confocal scanning ophthalmoscopy technology to test hypothesis that HIV-seropositive patients without history of retinitis with a history of a low CD4 count are more likely to have damage to their retinal nerve fiber layer (RNFL) when compared to patients with high CD4 count. In addition, we compared optic disc morphologic changes with glaucoma.

**Design:**

Cross-sectional study.

**Participants and Controls:**

171 patients were divided into four groups. The control group consisted of 40 eyes of 20 HIV-seronegative patients. The second group consisted of 80 eyes of 41 HIV-positive patients whose CD4 cell count never dropped below 100 (1.0 x 10^9^/L). The third group consisted of 44 eyes of 26 HIV-positive patients with a history of low CD4 counts <100. Fourth group consisted of 79 eyes of 79 patients with confirmed glaucoma who served as positive controls.

**Testing:**

Confocal scanning laser ophthalmoscopy was performed with the Heidelberg Retina Tomograph (HRT3) and data were analyzed with HRT3, software (Heyex version 1.5.10.0).

**Main Outcome Measures:**

Disc area, cup area, cup volume, rim volume, mean cup depth, maximum cup depth, cup-to-disc ration, mean RNFL thickness, and RNFL cross-sectional area.

**Results:**

Analysis of the global optic nerve and cup parameters showed no difference in disk area among the four groups. There was also no difference in cup, rim volume, mean cup depth, or maximum cup depth among the first three groups but they were all different from glaucoma group. The RNFL was thinner in glaucoma and both HIV-positive groups compared to HIV-seronegative subjects. The cross sectional RNFL area was thinner in both high and low CD4 HIV-positive groups compared to HIV-seronegative group in the nasal and temporal/inferior sectors, respectively. Glaucoma group showed thinning in all sectors.

**Conclusions:**

HIV retinopathy results in retinal nerve fiber layer loss without structural optic nerve supportive tissue change. RNFL damage may occur early in HIV disease by mechanism different than in glaucoma.

## Introduction

Human Immunodeficiency Virus (HIV) continues to be a major public health problem in the United States with 56,000 new HIV infections per year [1]. Worldwide, the HIV pandemic continues with 33 million infected people and an annual infection rate of 2.7 million with 2 million deaths. The current prevalence of HIV infection in the United States is estimated to be over 1.1 million [2]. Since 1996, with the advent of potent highly active antiretroviral therapy (HAART), we now see a significantly prolonged time between HIV infection and progression to acquired immunodeficiency syndrome (AIDS) and death in the United States [3] [4].

Treatment of HIV infection with highly active combination antiretroviral therapy has increased survival and shifted the spectrum of HIV-associated morbidity and mortality from opportunistic infections toward a variety of other medical conditions [5]. There is a cumulative effect of ongoing retinovascular disease that can be noted on clinical exam, including retinal nerve fiber layer (RNFL) infarcts [6] [7]. We have shown that even in the HAART-era patients with HIV suffer vision loss [8]. Our driving simulation studies showed that HIV patients had more driving errors despite normal visual acuity. Furthermore, the driving errors correlated with reduced retinal nerve fiber layer thickness [9]. We have hypothesized that the documented retinovascular disease which results in retinal cotton wool spots, retinal hemorrhages and non perfusion likely leads to cumulative death of retinal ganglion cells and lesions in the retinal nerve fiber layer [10].

Modern imaging has been instrumental in demonstrating retinal damage, in particular nerve layer loss in HIV patients without retinitis [11] [12]. Optical coherence tomography (OCT) and scanning laser polarimetric studies of the peripapillary RNFL show evidence of inner retinal damage, which seems cumulative in HIV patients without any evidence of infectious retinitis [13,14]. Our own studies have shown visual dysfunction in these patients, which has been confirmed by a major multicenter observational clinical trial group [15]. We and others have documented inner retinal damage which results in RNFL loss in OCT and scanning laser polarimetry [13,14] [16]. Other groups have demonstrated inner retinal damage using mfERG, visual field loss and focal ERG [17].

The Heidelberg Retina Tomograph (HRT) technology obtains topographic imaging of the optic disc and peripapillary retina including RNFL. The HRT has been widely investigated to assess the reproducibility of topographic measures and their clinical validity in differentiating normal from glaucomatous optic disks [18,19] [20]. Previously, we have shown the use of the HRT 2 in documenting damage of the RNFL in HIV patients both with and without retinitis [21]. This was a preliminary study using an earlier version of the sophisticated instrumentation that is available currently. In previous studies, the HRT software also defined the normative database based upon subjects with European ancestry a limited range of disc area and refractive corrections [22,23]. The HRT 3 is an updated imaging technology that now includes an expanded normative database including subjects of European, African, and Indian ancestry. In this study we look at the RNFL of HIV patients with different immune status using the advanced software (HRT 3). HRT 3 software has improved diagnostic ability compared with its predecessor [23] and we hypothesize that it may be possible to more accurately show optic disc changes and RNFL thinning with this advancement in software.

We wished to determine if the HIV related RNFL loss, previously demonstrated by OCT and scanning laser polarimetry, results in optic disc topography change. In glaucoma, optic disc cupping and excavation occurs along with RNFL loss. The amount of RNFL loss was most akin to early glaucoma in its severity and we wished to determine whether it was associated with changes in optic nerve topography and also to determine which manifestations of HRT changes are most sensitive to HIV disease.

## Methods

### Ethics Statement

Patients were enrolled in an Institutional Review Board (University of California San Diego Human Research Protection Program) approved study of HIV disease and written informed consent for imaging and data collection was obtained from the patients. Patients with positive serology for HIV were recruited from the University of California, San Diego AIDS Ocular Research Unit at the Jacobs Retina Center in La Jolla, California. Glaucoma patients were recruited from the University of California, San Diego Hamilton Glaucoma Center. Non-HIV volunteers were recruited as well for age-matched controls from hospital staff and family and friends of HIV-seropositive subjects. Patients had no history of other ocular disease or surgery other than diagnosis of glaucoma in our glaucoma population.

### Participants

The study included 260 eyes of 171 patients (114 men and 57 women). Patients were divided into four groups. The control group consisted of 40 eyes of 20 HIV-seronegative patients who served as control subjects. The mean age in this group was 38±10.6 years. The second group consisted of 80 eyes of 41 HIV-positive patients. We defined this group as our high CD4 group. We reviewed their medical records provided by their primary care doctor and laboratory and their CD4 cell count never dropped below 100 (1.0 x 10^9^/L). The mean age in this group was 40±6.6 years. The third group consisted of 44 eyes of 26 HIV-positive patients and was defined as our low CD4 group. Upon review of their medical records they had an episode with their CD4 level counts dropping below 100 at some point of time lasting for at least 6 months. Their mean age was 42±4.8 years. All HIV patients were treated with HAART therapy at the time of the examination. The fourth group was a positive control group without HIV consisting of patients with glaucomatous disease confirmed by clinical examination and visual field testing. The patients were diagnosed as having early or moderate defect based on Humphrey Visual Field mean deviation with an average MD of -5.3 dB (range 0.44 to -9.68 db).

Exclusion criteria in the first three groups included concurrent or healed CMV retinitis in an eye, previous ocular disease such as glaucoma (intraocular pressure > 21 mmHg, glaucomatous visual field defects on repeated testing), high myopia (>-5.0 diopters) and vision not refracted to at least 20/32. However, fellow eyes of CMV retinitis were included and automatically put to low CD4 group due to the high correlation of CMV retinitis and low CD4 count.

### Imaging studies

Confocal scanning laser ophthalmoscopy was performed with the Heidelberg Retina Tomograph (HRT3) and data were analyzed with HRT III software (Heyex version 1.5.10.0, HRTS viewer version 3.1.2.0, Heidelberg Engineering GmbH, Heidelberg, Germany). The HRT creates a three-dimensional map of the posterior field of view including the optic nerve head and adjacent retina by obtaining multiple optical sections at different depths using a confocal aperture. The details of the instrument have been previously described. [23,24]. In brief, the HRT scans the fundus with a 670-nm diode laser at a pixelation of 384x384 pixel in the retinal plane and records up to 64 partially overlapping confocal images over a 4 mm depth range. The default retinal reference plane in the Heidelberg Explorer software is located 50 μm posterior to the temporal disc margin. The following parameters, as calculated with HRT 3 software, were examined: disc area, cup area, cup volume, rim volume, mean cup depth, maximum cup depth, cup-to-disc ration, mean RNFL thickness, and RNFL cross-sectional area.

### Statistics

All analyses were conducted using SAS software version 9.2 (SAS, Inc., Cary, NC). We performed multivariate regression analysis to specifically take age and ethnicity into account and adjust for it. The level of statistical significance was set at p<0.05.

## Results

The study included 171 patients, of which 78 (45%) were Caucasians, 36 (21%) Hispanics, 51 (30%) African Americans and 6 (4%) Asians. The baseline characteristics are show in Table 1.

**Table 1:** Baseline characteristics of the four groups.

	Seronegative	HIV+ high CD4	HIV+ low CD4	Glaucoma
Male / female eyes	26 / 14	72 / 8	40 / 4	42 / 36
Age	38.1±10.6	40±6.6	42±4.8	70.6±10.2

The analysis of the global optic nerve and cup parameter (non-localized to the sectors {temporal, temporal/superior, temporal/inferior, nasal, nasal/superior, nasal/inferior}) showed no difference in cup, disk or rim areas between the three groups. The cup and rim areas were different in glaucoma group. There was also no difference in cup, rim volume, mean cup depth, or maximum cup depth between the three groups but the difference was when compared to glaucoma group. The RNFL, however, was thinner in the glaucoma group and in both groups with HIV-seropositive patients than in the group with HIV-seronegative subjects (Table 2). There was no significant difference between the two groups of HIV seropositive subjects (Table 2). Similarly, the RNFL area was reduced in glaucoma eyes and high and low CD4 groups as compared to the seronegative controls. However, there was also no difference in RNFL area between the low and high CD4 group. Looking at the six sectors this thinning in the mean RNFL was observed between both high (p = 0.044, p = 0.029, p = 0.045) and low (p = 0.048, p = 0.022, p = 0.049) CD4 groups and HIV-seronegative group in the nasal, nasal/inferior and temporal/inferior sectors, respectively (Table 3). Glaucoma group showed difference in all sectors compared to HIV-seronegative control group. The cross sectional RNFL area was thinner in both high (p = 0.030, p = 0.051) and low (p = 0.021, p = 0.048) CD4 group compared to HIV-seronegative group in nasal and temporal/inferior sectors, respectively. Again, glaucoma eyes showed thinning in all sectional areas compared to HIV-seronegative controls (Table 4).

**Table 2:** Independent, non-derived stereometric parameters of the optic nerve and nerve fiber layer in HIV+ patients compared to HIV-seronegative controls.

	HIV+ high CD4	P-value (HIV+ high to seronegative)	HIV+ low CD4	P-value (HIV+ low to seronegative)	HIV- seronegative control	P-value (Glaucoma to seronegative)	Glaucoma controls
	Mean ± SD (range)		Mean ± SD (range)		Mean ± SD (range)		Mean ± SD (range)
Disc area [mm2]	2.064±0.329 **(1.27–2.83)**	0.583	2.070±0.345 (1.43–3.21)	0.677	2.096±0.290 (1.64–2.61)	0.786	2.074±0.496 (1.07–3.77)
Cup area [mm2]	0.371±0.309 (0.00–1.56)	0.634	0.320±0.259 (0.00–0.96)	0.586	0.346±0.228 (0.00–0.84)	**3.868 E-12** *	0.961±0.490 (0.07–2.20)
Rim area [mm2]	1.692±0.312 (0.58–2.66)	0.314	1.750±0.429 (0.86–3.16)	0.992	1.749±0.289 (1.32–2.42)	**1.279 E-18** *	1.113±0.330 (0.52–2.48)
Cup volume [mm3]	0.069±0.089 (0.00–0.49)	0.978	0.057±0.067 (0.00–0.26)	0.383	0.069±0.068 (0.00–0.23)	**1.729 E-8** *	0.295±0.239 (0.00–1.02)
Rim volume [mm3]	0.452±0.176 (0.09–1.14)	0.187	0.478±0.212 (0.09–1.08)	0.658	0.495±0.167 (0.21–0.89)	**5.602 E-15** *	0.251±0.126 (0.07–0.69)
Mean cup depth [mm]	0.159±0.073 (0.03–0.48)	0.564	0.149±0.076 (0.03–0.38)	0.222	0.166±0.064 (0.03–0.31)	**3.818 E-10** *	0.313±0.132 (0.09–0.65)
Max cup depth [mm]	0.486±0.199 (0.09–1.03)	0.336	0.442±0.218 (0.08–1.05)	0.056	0.522±0.200 (0.08–0.85)	**4.496 E-06** *	0.728±0.236 (0.31–1.44)
Mean RNFL thickness [mm]	0.229±0.094 (-0.29–0.45)	**0.028** *	0.234±0.080 (0.08–0.58)	**0.039** *	0.265±0.064 (0.15–0.40)	**1.566 E-08** *	0.185±0.071 (0.02-.037)
RNFL cross sectional area [mm2]	1.167±0.489 (-1.47–2.58)	**0.022** *	1.189±0.386 (0.37–2.51)	**0.020** *	1.358±0.335 (0.76–2.05)	**7.575 E-09** *	0.932±0.369 (0.13–1.98)

HIV+ = subjects who are human immunodeficiency virus (HIV) positive, HIV- = subjects who are HIV negative controls, CD4 = CD4 cell count, SD = standard deviation, RNFL = retinal nerve fiber layer, mm–millimeter.

*—statistically significant

**Table 3:** Variability of HRT3 mean retinal nerve fiber layer thickness by retinal regions among HIV+ and HIV-seronegative patients.

	HIV+ high CD4	P-value (HIV+ high to seronegative)	HIV+ low CD4	P-value (HIV+ low to seronegative)	HIV- seronegative control
Nasal	0.257±0.131 (-0.52–0.51)	**0.044** *	0.264±0.112 (0.04–0.69)	**0.048** *	0.303±0.100 (0.08–0.48)
Nasal/Sup	0.310±0.148 (-0.53–0.81)	0.628	0.325±0.130 (0.01–0.88)	0.649	0.346±0.089 (0.17–0.55)
Nasal/Inf	0.336±0.126 (-0.22–0.68)	**0.029** *	0.329±0.100 (0.10–0.65)	**0.022** *	0.383±0.099 (0.22–0.63)
Temp/Inf	0.250±0.098 (-0.04–0.49)	**0.045** *	0.246±0.099 (-0.01–0.59)	**0.049** *	0.290±0.092 (0.14–0.49)
Temporal	0.083±0.032 (-0.05–0.18)	0.334	0.086±0.024 (0.04–0.20)	0.213	0.096±0.027 (0.04–0.15)
Temp/Sup	0.261±0.127 (-0.39–0.64)	0.421	0.268±0.101 (0.09–0.73)	0.442	0.302±0.083 (0.16–0.52)

HRT = Heidelberg retina tomography, HIV+ = subjects who are human immunodeficiency virus (HIV) positive, HIV- = subjects who are HIV negative controls, CD4 = CD4 cell count, SD = standard deviation, RNFL = retinal nerve fiber layer, Sup = superior, Inf = inferior.

*—statistically significant

**Table 4:** Variability of HRT3 retinal nerve fiber layer cross sectional area by retinal regions among HIV+ and HIV-seronegative patients.

	HIV+ high CD4	P-value (HIV+ high to sero-negative)	HIV+ low CD4	P-value (HIV+ low to sero-negative)	HIV- seronegative control
Nasal	0.319±0.167 (-0.65–0.71)	**0.030** *	0.324±0.127 (0.04–0.74)	**0.021** *	0.378±0.122 (0.09–0.58)
Nasal/Sup	0.202±0.100 (-0.36–0.54)	0.641	0.215±0.086 (0.01–0.50)	0.702	0.228±0.061 (0.11–0.40)
Nasal/Inf	0.217±0.082 (-0.14–0.44)	0.120	0.213±0.066 (0.06–0.37)	0.567	0.248±0.071 (0.14–0.43)
Temp/Inf	0.165±0.066 (-0.02–0.36)	0.051	0.163±0.066 (-0.01–0.33)	**0.048** *	0.193±0.068 (0.08–0.34)
Temporal	0.103±0.041 (-0.06–0.26)	0.596	0.107±0.030 (0.04–0.21)	0.611	0.120±0.034 (0.05–0.19)
Temp/Sup	0.170±0.085 (-0.26–0.43)	0.552	0.173±0.064 (0.06–0.39)	0.584	0.197±0.060 (0.09–0.34)

HRT = Heidelberg retina tomography, HIV+ = subjects who are human immunodeficiency virus (HIV) positive, HIV- = subjects who are HIV negative controls, CD4 = CD4 cell count, SD = standard deviation, RNFL = retinal nerve fiber layer, Sup = superior, Inf = inferior.

*—statistically significant

## Discussion

For over 20 years our research group has pioneered investigations into vision loss and dysfunction in patients with HIV with and without retinitis. We have found that in the era of highly active antiretroviral therapy (HAART), vision is not normal in HIV patients [references]. Despite the presence of effective treatment for patients with HIV research from all over the world confirms that HIV-associated neurocognitive disorders are common among the patients [25]. We have demonstrated that we can document retinal damage due to cotton wool spots (CWS) as long as 18 years after the active phase [26]. The RNFL, inner plexiform layer (IPL), and the inner nuclear layer (INL) exhibited decreased thickness while the outer nuclear layer (ONL) demonstrated a significant increase. Using structural imaging test we found that patients with HIV had significant decrease in RNFL thickness [14] [13]. Although there is evidence of RNFL loss in patients with HIV, nobody has evaluated whether this changes the optic nerve head. Our goal was to study if RNFL loss was associated with optic disk changes to more fully characterize this inner retinal disease that does not cause vision loss.

Imaging modalities provide adjunctive information to the clinical exam in assessing damage to the RNFL and optic disc. The HRT3 is an ideal imaging instrument to quantify the three-dimensional structure of the optic nerve head. It has a very high topographic accuracy in detecting small changes to the shape of the optic nerve head due to the large number of image points in space (over 9 million voxels; 64 slices by 384 x-pixel by 384 y-pixel) [27] [28]. In this study, we used our own database of seronegative and glaucoma controls to improve the robustness of our study. In glaucoma, an optic neuropathy with loss of the RNFL, clinically detectable nerve fiber atrophy often precedes the onset of glaucomatous field loss [29]. Structural changes of the optic disc and RNFL recognition are important clinically for the early diagnosis of the disease [30]. Similarly, in HIV patients it is important to be able to morphologically detect changes in the optic disc and RNFL early. Our results indicate that the RNFL was damaged in some areas. This is a validation of our study design.

We evaluated optic disc topography for changes that are characteristic for glaucoma, and found no pattern suggesting glaucomatous optic neuropathy in the HIV population. The measurements included horizontal and vertical cup to disk ratio, or disk, cup and rim area ratios. This was observed in the global parameter analysis (Table 2) as well as in sector analysis (not shown). A previous study by Besada et al. [31] using the HRT 2 also failed to demonstrate changes in optic nerve parameters in HIV+ patients compared to HIV-seronegative controls. Our study has the advantage of using improved HRT technology and a larger number of patients including a positive control group of glaucomatous eyes. Our study also validates the loss of RNFL in the HIV population.

In the studied population we found a defect in the mean RNFL and cross-sectional RNFL area in the nasal, nasal/inferior, temporal/inferior and in nasal and temporal/inferior sectors, respectively. In our previous study with time domain OCT technology, the defect was shown to affect temporal, superior and inferior quadrants but not nasal quadrant [13]. A study by OCT in children agreed with this distribution of damage but found also thinning of nasal RNFL [32]. Scanning laser polarimetry with variable corneal compensation localized this defect to superior and inferior areas rather than lateral locations. [14]. Because each of the instruments used to image the RNFL use distinct optical methods, it may be difficult to compare them directly. Correlation of these in vivo findings and histopathologic evaluations could reveal the exact location of HIV-related RNFL damage. Recent histopathological evaluation of patients with HIV found not only inner retinal layer damage, but also undetected outer retinal layer damage especially photoreceptors and retinal pigment epithelium [33].

In this study, there were no significant optic disc morphologic changes that are seen in glaucoma, such as cupping, were observed in patients with HIV disease. One would assume this to be very likely due to excluding glaucomatous eyes thereby introducing selection bias. In spite of that, however, we still have found a significant loss of RNFL thickness in these patients biasing not for but against the hypothesis and making our results more convincing. We have also shown an imaging modality (HRT 3) as a means of identifying the amount of damage in patients suffering from HIV-related RNFL thinning.

Interestingly, we found no significant difference in RNFL thickness between the high and low CD4 groups. This observation is difficult to explain but points to the fact that also high CD4 HIV-positive patients shows some structural damage despite their immune reconstitution. There was also no evidence of glaucomatous optic cupping, glaucomatous optic neuropathy or optic cup changes between the HIV+ groups and seronegative controls. This conclusion is based on cross-sectional primary measurements of the cup and disc, not based on derived measurements. Our findings confirm that HIV related inner retinal damage and RNFL loss does not result in measurable changes in the optic nerve head morphology other than loss of RNFL. Eyes with HIV-related damage experience a reduction in RNFL thickness but no glaucoma-like appearance of the optic nerve head. This suggests that HIV retinopathy is a unique entity that does not result in structural changes to the optic nerve supportive tissue, however it is associated with vision loss as measured by subjective measures such as visual field testing, color vision test, and multifocal electroretinography [11] [34]. Several reports have demonstrated loss of RNFL without optic disc changes in other retinovascular diseases such as diabetic retinopathy [35] [36] [37]. Longitudinal studies of RNFL loss and optic cup parameters would help determine the natural history of this condition and maybe optic disc changes with time. In addition, ganglion cell measurement analysis may be helpful for detecting early HIV-related retinopathy. Current OCT segmentation software allows ganglion cell layer thickness analysis [38].

In summary, we have shown that HIV retinopathy results in retinal nerve fiber layer loss without structural optic nerve supportive tissue changes. This differentiates it from glaucoma. RNFL damage may occur early in HIV disease and the mechanism is different than glaucoma. After years of seropositivity, there are no measurable optic nerve head changes other than RNFL loss. Testing in clinics with confocal tomography or other measures of optic nerve head topography may be additionally useful in ruling out early glaucoma in patients with HIV who have loss of RNFL.

## References

[1] CDC (2012) Estimated HIV incidence in the United States, 2007–2010.

[2] CDC (2012) Monitoring selected national HIV prevention and care objectives by using HIV surveillance data—United States and 6 U.S. dependent areas—2010.

[3] Palella FJ, Delaney KM, Moorman AC, Loveless MO, Fuhrer J, Satten GA, et al (1998) Declining morbidity and mortality among patients with advanced human immunodeficiency virus infection. HIV Outpatient Study Investigators. N Engl J Med 338: 853–860. 9516219 10.1056/NEJM199803263381301

[4] Bhaskaran K, Hamouda O, Sannes M, Boufassa F, Johnson AM, Lambert PC, et al (2008) Changes in the risk of death after HIV seroconversion compared with mortality in the general population. JAMA 300: 51–59. 10.1001/jama.300.1.51 18594040

[5] Vellozzi C, Brooks JT, Bush TJ, Conley LJ, Henry K, Carpenter CC, et al (2009) The study to understand the natural history of HIV and AIDS in the era of effective therapy (SUN Study). Am J Epidemiol 169: 642–652. 10.1093/aje/kwn361 19074775

[6] Freeman WR, Henderly DE, Lipson BK, Rao NA, Levine AM (1989) Retinopathy before the diagnosis of AIDS. Annals of Ophthalmology 21: 468–474. 2560616

[7] Jabs DA, Green WR, Fox R, Polk BF, Bartlett JG (1989) Ocular manifestations of acquired immune deficiency syndrome. Ophthalmology 96: 1092–1099. 2549483 10.1016/s0161-6420(89)32794-1

[8] Sample PA, Plummer DJ, Mueller AJ, Matsubara KI, Sadun A, Grant I, et al (1999) Pattern of early visual field loss in HIV-infected patients. Arch Ophthalmol 117: 755–760. 10369585 10.1001/archopht.117.6.755

[9] Cheng S, Klein H, Bartsch DU, Kozak I, Marcotte TD, Freeman WR (2011) Relationship between retinal nerve fiber layer thickness and driving ability in patients with human immunodeficiency virus infection. Graefes Arch Clin Exp Ophthalmol 249: 1643–1647. 10.1007/s00417-011-1735-4 21732109 PMC4144406

[10] Kozak I, Bartsch DU, Cheng L, Freeman WR (2007) Hyperreflective sign in resolved cotton wool spots using high-resolution optical coherence tomography and optical coherence tomography ophthalmoscopy. Ophthalmology 114: 537–543. 17324696 10.1016/j.ophtha.2006.06.054

[11] Kozak I, Sample PA, Hao J, Freeman WR, Weinreb RN, Lee TW, et al (2007) Machine learning classifiers detect subtle field defects in eyes of HIV individuals. Trans Am Ophthalmol Soc 105: 111–118; discussion 119–120. 18427600 PMC2258123

[12] Falkenstein IA, Bartsch DU, Azen SP, Dustin L, Sadun AA, Freeman WR (2008) Multifocal electroretinography in HIV-positive patients without infectious retinitis. Am J Ophthalmol 146: 579–588. 10.1016/j.ajo.2007.12.021 18280451 PMC2614872

[13] Kozak I, Bartsch DU, Cheng L, Kosobucki BR, Freeman WR (2005) Objective analysis of retinal damage in HIV-positive patients in the HAART era using OCT. Am J Ophthalmol 139: 295–301. 15733991 10.1016/j.ajo.2004.09.039 PMC3757251

[14] Kozak I, Bartsch DU, Cheng L, McCutchan A, Weinreb RN, Freeman WR (2007) Scanning laser polarimetry demonstration of retinal nerve fiber layer damage in human immunodeficiency virus-positive patients without infectious retinitis. Retina 27: 1267–1273. 18046236 10.1097/IAE.0b013e31806463fb

[15] Freeman WR, Van Natta ML, Jabs D, Sample PA, Sadun AA, Thorne J, et al (2008) Vision function in HIV-infected individuals without retinitis: report of the Studies of Ocular Complications of AIDS Research Group. Am J Ophthalmol 145: 453–462. 10.1016/j.ajo.2007.10.013 18191094 PMC2359724

[16] Kalyani PS, Holland GN, Fawzi AA, Arantes TE, Yu F, Sadun AA, et al (2012) Association between retinal nerve fiber layer thickness and abnormalities of vision in people with human immunodeficiency virus infection. Am J Ophthalmol 153: 734–742, 742 e731. 10.1016/j.ajo.2011.09.019 22245459 PMC4121665

[17] Iragui VJ, Kalmijn J, Plummer DJ, Sample PA, Trick GL, Freeman WR (1996) Pattern electroretinograms and visual evoked potentials in HIV infection: evidence of asymptomatic retinal and postretinal impairment in the absence of infectious retinopathy. Neurology 47: 1452–1456. 8960726 10.1212/wnl.47.6.1452

[18] Iester M, Mikelberg FS, Courtright P, Burk RO, Caprioli J, Jonas JB, et al (2001) Interobserver variability of optic disk variables measured by confocal scanning laser tomography. Am J Ophthalmol 132: 57–62. 11438054 10.1016/s0002-9394(01)00938-2

[19] Mikelberg FS, Wijsman K, Schulzer M (1993) Reproducibility of topographic parameters obtained with the heidelberg retina tomograph. J Glaucoma 2: 101–103. 19920494

[20] Miglior S, Albe E, Guareschi M, Rossetti L, Orzalesi N (2002) Intraobserver and interobserver reproducibility in the evaluation of optic disc stereometric parameters by Heidelberg Retina Tomograph. Ophthalmology 109: 1072–1077. 12045046 10.1016/s0161-6420(02)01032-1

[21] Plummer DJ, Bartsch DU, Azen SP, Max S, Sadun AA, Freeman WR (2001) Retinal nerve fiber layer evaluation in human immunodeficiency virus- positive patients. Am J Ophthalmol 131: 216–222. 11228298 10.1016/s0002-9394(00)00787-x

[22] Wollstein G, Garway-Heath DF, Hitchings RA (1998) Identification of early glaucoma cases with the scanning laser ophthalmoscope. Ophthalmology 105: 1557–1563. 9709774 10.1016/S0161-6420(98)98047-2

[23] De Leon-Ortega JE, Sakata LM, Monheit BE, McGwin G Jr., Arthur SN, Girkin CA (2007) Comparison of diagnostic accuracy of Heidelberg Retina Tomograph II and Heidelberg Retina Tomograph 3 to discriminate glaucomatous and nonglaucomatous eyes. Am J Ophthalmol 144: 525–532. 17693382 10.1016/j.ajo.2007.06.021 PMC3928044

[24] Bartsch DU, Intaglietta M, Bille JF, Dreher AW, Gharib M, Freeman WR (1989) Confocal laser tomographic analysis of the retina in eyes with macular hole formation and other focal macular diseases. American Journal of Ophthalmology 108: 277–287. 2476030 10.1016/0002-9394(89)90118-9

[25] Letendre SL, Ellis RJ, Ances BM, McCutchan JA (2010) Neurologic complications of HIV disease and their treatment. Top HIV Med 18: 45–55. 20516524 PMC5077300

[26] Gomez ML, Mojana F, Bartsch DU, Freeman WR (2009) Imaging of long-term retinal damage after resolved cotton wool spots. Ophthalmology 116: 2407–2414. 10.1016/j.ophtha.2009.05.012 19815278 PMC4172325

[27] Philippin H, Unsoeld A, Maier P, Walter S, Bach M, Funk J (2006) Ten-year results: detection of long-term progressive optic disc changes with confocal laser tomography. Graefes Arch Clin Exp Ophthalmol 244: 460–464. 16133021 10.1007/s00417-005-0094-4

[28] Lin D, Leung CK, Weinreb RN, Cheung CY, Li H, Lam DS (2009) Longitudinal evaluation of optic disc measurement variability with optical coherence tomography and confocal scanning laser ophthalmoscopy. J Glaucoma 18: 101–106. 10.1097/IJG.0b013e318179f879 19225344

[29] Sommer A, Katz J, Quigley HA, Miller NR, Robin AL, Richter RC, et al (1991) Clinically detectable nerve fiber atrophy precedes the onset of glaucomatous field loss. Arch Ophthalmol 109: 77–83. 1987954 10.1001/archopht.1991.01080010079037

[30] Badala F, Nouri-Mahdavi K, Raoof DA, Leeprechanon N, Law SK, Caprioli J (2007) Optic disk and nerve fiber layer imaging to detect glaucoma. Am J Ophthalmol 144: 724–732. 17868631 10.1016/j.ajo.2007.07.010 PMC2098694

[31] Besada E, Shechtman D, Black G, Hardigan PC (2007) Laser scanning confocal ophthalmoscopy and polarimetry of human immunodeficiency virus patients without retinopathy, under antiretroviral therapy. Optom Vis Sci 84: 189–196. 17435532 10.1097/OPX.0b013e31803399f3

[32] Moschos MM, Mostrou G, Psimenidou E, Spoulou V, Theodoridou M (2007) Objective analysis of retinal function in HIV-positive children without retinitis using optical coherence tomography. Ocul Immunol Inflamm 15: 319–323. 17763130 10.1080/09273940701375154

[33] Kozak I, Sasik R, Freeman WR, Sprague LJ, Gomez ML, Cheng L, et al (2013) A Degenerative Retinal Process in HIV-Associated Non-Infectious Retinopathy. PLoS One 8: e74712 10.1371/journal.pone.0074712 24069333 PMC3775801

[34] Goldbaum MH, Falkenstein I, Kozak I, Hao J, Bartsch DU, Sejnowski T, et al (2008) Analysis with support vector machine shows HIV-positive subjects without infectious retinitis have mfERG deficiencies compared to normal eyes. Trans Am Ophthalmol Soc 106: 196–204; discussion 204–195. 19277235 PMC2646437

[35] Lopes de Faria JM, Russ H, Costa VP (2002) Retinal nerve fibre layer loss in patients with type 1 diabetes mellitus without retinopathy. Br J Ophthalmol 86: 725–728. 12084737 10.1136/bjo.86.7.725 PMC1771182

[36] Ozdek S, Lonneville YH, Onol M, Yetkin I, Hasanreisoglu BB (2002) Assessment of nerve fiber layer in diabetic patients with scanning laser polarimetry. Eye (Lond) 16: 761–765. 12439673 10.1038/sj.eye.6700207

[37] van Dijk HW, Kok PH, Garvin M, Sonka M, Devries JH, Michels RP, et al (2009) Selective loss of inner retinal layer thickness in type 1 diabetic patients with minimal diabetic retinopathy. Invest Ophthalmol Vis Sci 50: 3404–3409. 10.1167/iovs.08-3143 19151397 PMC2937215

[38] Garvin MK, Lee K, Burns TL, Abramoff MD, Sonka M, Kwon YH (2013) Reproducibility of SD-OCT-based ganglion cell-layer thickness in glaucoma using two different segmentation algorithms. Invest Ophthalmol Vis Sci 54: 6998–7004. 10.1167/iovs.13-12131 24045993 PMC3809946

